# Identification, Replication, and Functional Fine-Mapping of Expression Quantitative Trait Loci in Primary Human Liver Tissue

**DOI:** 10.1371/journal.pgen.1002078

**Published:** 2011-05-26

**Authors:** Federico Innocenti, Gregory M. Cooper, Ian B. Stanaway, Eric R. Gamazon, Joshua D. Smith, Snezana Mirkov, Jacqueline Ramirez, Wanqing Liu, Yvonne S. Lin, Cliona Moloney, Shelly Force Aldred, Nathan D. Trinklein, Erin Schuetz, Deborah A. Nickerson, Ken E. Thummel, Mark J. Rieder, Allan E. Rettie, Mark J. Ratain, Nancy J. Cox, Christopher D. Brown

**Affiliations:** 1 Cancer Research Center, Committee on Clinical Pharmacology and Pharmacogenomics, Department of Medicine, The University of Chicago, Chicago, Illinois, United States of America; 2 Department of Genome Sciences, University of Washington, Seattle, Washington, United States of America; 3 Section of Genetic Medicine, Department of Medicine, The University of Chicago, Chicago, Illinois, United States of America; 4 Section of Hematology/Oncology, Department of Medicine, The University of Chicago, Chicago, Illinois, United States of America; 5 Department of Medicinal Chemistry, School of Pharmacy, University of Washington, Seattle, Washington, United States of America; 6 Department of Pharmaceutics, University of Washington, Seattle, Washington, United States of America; 7 Merck Research Laboratories, Boston, Massachusetts, United States of America; 8 SwitchGear Genomics, Menlo Park, California, United States of America; 9 Department of Pharmaceutical Sciences, St. Jude Children's Research Hospital, Memphis, Tennessee, United States of America; 10 Institute for Genomics and Systems Biology, The University of Chicago and Argonne National Laboratory, Chicago, Illinois, United States of America; 11 Departments of Human Genetics and Ecology and Evolution, The University of Chicago, Chicago, Illinois, United States of America; Princeton University, United States of America

## Abstract

The discovery of expression quantitative trait loci (“eQTLs”) can help to unravel genetic contributions to complex traits. We identified genetic determinants of human liver gene expression variation using two independent collections of primary tissue profiled with Agilent (n = 206) and Illumina (n = 60) expression arrays and Illumina SNP genotyping (550K), and we also incorporated data from a published study (n = 266). We found that ∼30% of SNP-expression correlations in one study failed to replicate in either of the others, even at thresholds yielding high reproducibility in simulations, and we quantified numerous factors affecting reproducibility. Our data suggest that drug exposure, clinical descriptors, and unknown factors associated with tissue ascertainment and analysis have substantial effects on gene expression and that controlling for hidden confounding variables significantly increases replication rate. Furthermore, we found that reproducible eQTL SNPs were heavily enriched near gene starts and ends, and subsequently resequenced the promoters and 3′UTRs for 14 genes and tested the identified haplotypes using luciferase assays. For three genes, significant haplotype-specific *in vitro* functional differences correlated directly with expression levels, suggesting that many *bona fide* eQTLs result from functional variants that can be mechanistically isolated in a high-throughput fashion. Finally, given our study design, we were able to discover and validate hundreds of liver eQTLs. Many of these relate directly to complex traits for which liver-specific analyses are likely to be relevant, and we identified dozens of potential connections with disease-associated loci. These included previously characterized eQTL contributors to diabetes, drug response, and lipid levels, and they suggest novel candidates such as a role for *NOD2* expression in leprosy risk and *C2orf43* in prostate cancer. In general, the work presented here will be valuable for future efforts to precisely identify and functionally characterize genetic contributions to a variety of complex traits.

## Author Summary

Many disease-associated genetic variants do not alter protein sequences and are difficult to precisely identify. Discovery of expression quantitative trait loci (eQTL), or correlations between genetic variants and gene expression levels, offers one means of addressing this challenge. However, eQTL studies in primary cells have several shortcomings. In particular, their reproducibility is largely unknown, the variables that generate unreliable associations are uncharacterized, and the resolution of their findings is constrained by linkage disequilibrium. We performed a three-way replication study of eQTLs in primary human livers. We demonstrated that ∼67% of cis-eQTL associations are replicated in an independent study and that known polymorphisms overlapping expression probes, SNP-to-gene distance, and unmeasured confounding variables all influence the replication rate. We fine-mapped 14 eQTLs and identified causative polymorphisms in the promoter or 3′UTR for 3 genes, suggesting that a considerable fraction of eQTLs are driven by proximal variants that are amenable to functional isolation. Finally, we found hundreds of overlaps between SNPs associated with complex traits and replicated eQTL SNPs. Our data provide both cautionary (*i.e.* non-reproducibility of many strong eQTLs) and optimistic (*i.e.* precise identification of functional non-coding variants) forecasts for future eQTL analyses and the complex traits that they influence.

## Introduction

Genome-wide association studies have uncovered numerous robust associations between common variants and complex traits, but only a minority of these can be traced to protein-altering polymorphisms [1]. It is likely that most of these associations result from non-coding variants. One hypothesis is that such variants modify cis-regulatory sequences and thereby change the expression levels of one or more target genes. Variance in gene expression plays essential roles in numerous important processes and is highly heritable in human populations [2].

Considering this, the discovery of genetic variants that have a functional impact on gene expression is a potentially powerful means to facilitate more accurate and robust identification of associations between variants and disease. Such discoveries may also provide mechanistic insight into otherwise anonymous genotype-phenotype correlations that often span many correlated variants across multiple genes. In large part due to this potential there has been recent substantial interest in the identification of expression quantitative trait loci (eQTLs) [3]–[10].

Regulation of gene expression in the liver is of particular interest given its vital roles in maintaining homeostasis and health, including synthesis of most essential serum proteins, the production of bile and its carriers, and the regulation of nutrients. The liver is also the predominant organ in xenobiotic metabolism, and it has been estimated that 75% of the 200 most widely prescribed drugs are eliminated from the body through liver metabolism [11]. Altered metabolism by genetic factors affects the systemic availability and residence time of xenobiotics and hence their toxic and pharmacologic effects [12].

While eQTL studies have made valuable contributions to genetic research (e.g., [13]), there exist several practical limitations to consider. First, most eQTL studies are conducted in immortalized, lymphoblastoid cell lines (LCLs), which clearly have utility for the interpretation of human disease associations, particularly with immunity-related phenotypes [14], [15]. However, the use of such cell lines potentially introduces artifacts associated with immortalization, subsequent passage, and growth conditions prior to harvest [16]. Second, eQTLs may exhibit spatiotemporal specificity [17], [18], presumably driven by polymorphisms located within tissue specific regulatory elements, and eQTL studies may be maximally informative for any given trait when conducted in a relevant, non-transformed cell type. Third, environmental factors and other, mostly hidden, confounding variables are known to significantly affect gene expression levels and measurements [19]–[23]. Fourth, most eQTL studies fail to provide replication on an independent set of samples with independent experimental assessment (see [24]–[27], [23] for exceptions).

We sought to address these limitations, and conducted two independent eQTL studies and compared these results to a third, published study. Genetic analyses were performed using Bayesian regression [28], [29] after controlling for age, sex, ancestry, and unmeasured confounding variables [20]. Using the UC liver panel as a ‘discovery’ cohort and the UW and Merck data as replication panels, we found that ∼30% of eQTLs identified at stringent thresholds failed to replicate in either of the two replication studies. We show that this is likely due to several factors, including SNPs in probes, but the effects of unmeasured confounding variables were particularly pronounced. We also found that reproducible eQTL associations were enriched near proximal promoters and 3′ UTRs. Through targeted resequencing and luciferase experiments, we identified 3 significant haplotype-specific *in vitro* functional effects that directly support a liver eQTL. These data functionally validate the enrichment for eQTLs near gene ends and suggest that many eQTLs can be rapidly fine mapped to a causative variant or haplotype. Finally, given our study design we identified hundreds of genes with reproducible SNP-associated expression levels, a subset of which provide strong mechanistic hypotheses for published associations between SNPs and disease.

## Results

### Three independent sample collections

We analyzed two independent sets of primary liver tissues at the University of Chicago (UC; n = 206) and University of Washington (UW; n = 60). We genotyped both sets of samples using Illumina SNP arrays (quad-610 and 550 k for UC and UW, respectively); to improve mapping power [30], [28] and replication ascertainment, additional genotypes were imputed using HAPMAP reference genotype panels (see Methods). Gene expression levels were analyzed using Agilent (UC) and Illumina (UW) expression arrays. We considered the UC liver collection as a ‘discovery’ set and used as replication panels the UW collection and a published set of liver eQTL data (Merck; n = 266) [31]. However, we note that the conclusions drawn below were robust to the choice of a ‘discovery’ set (Figure S1). All samples analyzed across all three studies were unique. Microarray expression probes from both platforms were remapped to RefSeq gene models to aid in cross platform comparisons. A total of 14,703 RefSeq genes were surveyed in the UC reference study while 11,245 RefSeq genes were present on all three platforms. We have made these data and results publicly available through the GEO and SCAN databases (http://www.scandb.org/) [32].

### Demographic effects

**Figure 1:**
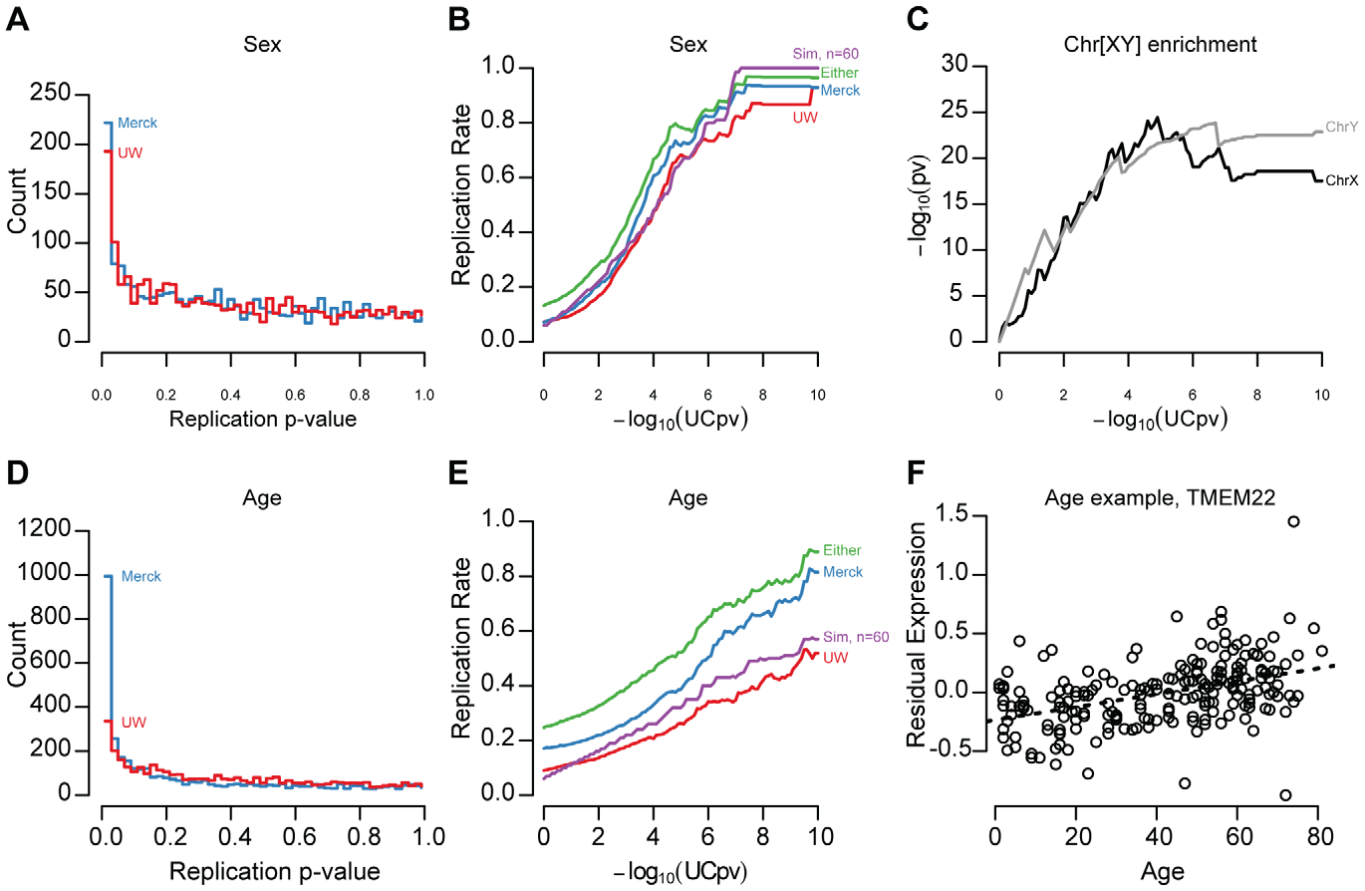
Age and sex effect replication. (A,D) Replication panel linear regression t-test p-values for genes with significant Sex (A) and Age (D) effects in the UC panel (<5% FDR) were binned (x-axis) and the number of genes per bin (y-axis) is displayed separately for the UW (red) and Merck (blue) replication sets. (B,E) The replication rate of sex (B) and age (E) associations is depicted as a function of UC p-value for UW (red), Merck (blue), either replication study (green), and for the n = 60 resampled data (purple). Note that, at more stringent significance thresholds, the replication rate increases but fewer genes are included. (C) Genes with significant sex associations are enriched on the X (black) and Y (grey) chromosomes. Hypergeometric test p-values (y-axis, log_10_ scaled) are plotted as a function of the discovery set effect significance threshold (x-axis, log_10_-scaled). (F) An example of a gene (*TMEM22*) whose expression level (y-axis) is associated with age (x-axis). Each point represents the expression level (y-axis), adjusted for surrogate variables, and age (x-axis) of an individual sample.

After correcting for technical effects and unmeasured confounding variables, we found that thousands of gene expression traits were significantly associated with demographic variables. At a 5% false discovery rate (FDR), 769, 336, and 3,110 genes were significantly associated with ancestry, sex, and age, respectively within the UC livers. Genes significantly affected by sex or age (FDR<5%, Figure 1, Figure S2, examples displayed in Figure S3) have a marked enrichment for small p-values in both replication samples (Figure 1A, 1D). To lessen the influence of differential statistical power among the three studies (n = 206, 60, 266), we defined ‘replication’ as having a nominally significant p-value in the independent sample (p-value<0.05) and having a concordant effect direction (*i.e.*, is YFG more highly expressed in males or females?). 29.9% and 32.1% of genes significantly affected by sex (UC sex t-test FDR<5%) replicated in the UW and Merck studies, respectively (Figure 1B). At more stringent thresholds, validation rates exceeded 80%, albeit with fewer included genes (Figure 1B). We also note that the sex-associated gene set was strongly enriched for genes on the X and Y chromosomes (Figure 1C; X chromosome, hypergeometric test, p-value = 1.72×10^−14^, Y chromosome, p-value<2×10^−16^), as would be expected for genes with sex-associated expression levels. Effects due to age were less reproducible: 13.2% and 21.5% of significantly age associated genes (UC age t-test FDR<5%) replicated in the UW and Merck studies, respectively (Figure 1E; an example of a replicated age-associated gene, *TMEM22*, is displayed in Figure 1F). Effect sizes for both sex and age were correlated across studies (Figure S4; Spearman's rho, UC-UW sex = 0.597, UC-Merck sex = 0.720, UC-UW age = 0.333, UC-Merck age = 0.159), underscoring the reproducibility of demographic effect estimates.

It is possible that both age and sex replication rates are downwardly biased due to differences in age and sex distributions (Table 1). To quantify the potential effects of heterogeneous sample sizes and unbalanced study designs, we performed resampling studies within the UC discovery cohort. Demographic effect replication rates were recalculated using 60 samples that were race, sex, and age (+/−3years) matched to the UW distribution (Figure 1B, 1E; see Methods). We found that 34% of sex effects and 15% of age effects replicated by simulation, supporting the conclusion that sample size and demographic heterogeneity do generate significant covariate associations that our replication studies are unable or underpowered to detect.

**Table 1:** Sample demographic summaries of all three studies.

		Study		
Category	Subcategory	University of Chicago	University of Washington	Merck
Final Sample #		206	60	266
Gender	Male	131	32	137
	Female	75	28	129
Age	25th percentile	21	28	40
	50th percentile	46	45	52
	75th percentile	59	55	62
Race	European-American	183	55	266
	Non-European	23	5	0
Genotyping platform	Name	Illumina 610 Quad	Illumina 550K v3	Affymetrix 500K; Illumina 650Y
	GEO accession	GPL8887	GPL6981	
Expression platform	Name	Agilent 4×44K	HumanRef-8 v.2	Agilent Custom
	GEO accession	GPL4133	GPL5060	GPL4371
Expression replicates	Mean	2.25	2	1
Fraction expression probes overlapping dbSNP130		0.274	0.191	NA
Data availability	GEO series	GSE26106	GSE26106	GSE9588
Publication		*this study*	*this study*	PMID18462017

### cis-eQTL mapping

**Figure 2:**
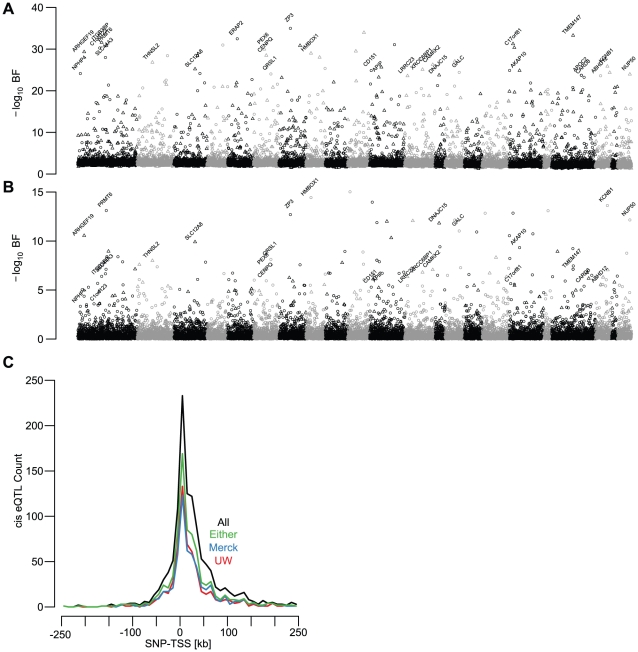
eQTL characteristics. (A,B) Manhattan plots depicting best associated cis-eQTLs for all gene expression traits in the UC (A) and UW (B) studies. Autosomes are ordered and alternately colored along the x-axis. BF of the SNP-gene pair is plotted on the y-axis. Probes overlapping common polymorphisms are plotted as triangles, probes without known SNPs are plotted as open circles. For display purposes, genes with UC BF>23 that replicate in the UW study are labeled with gene names. (C) Distribution of distances from each gene's best associated SNP to its TSS. Negative and positive values denote SNPs 5′ and 3′ of TSS, respectively. Data are plotted for all significant UC eQTLs (BF>5, black), eQTLs replicated in the UW (red), Merck (blue), and eQTLs replicated in either UW or Merck (green).

**Figure 3:**
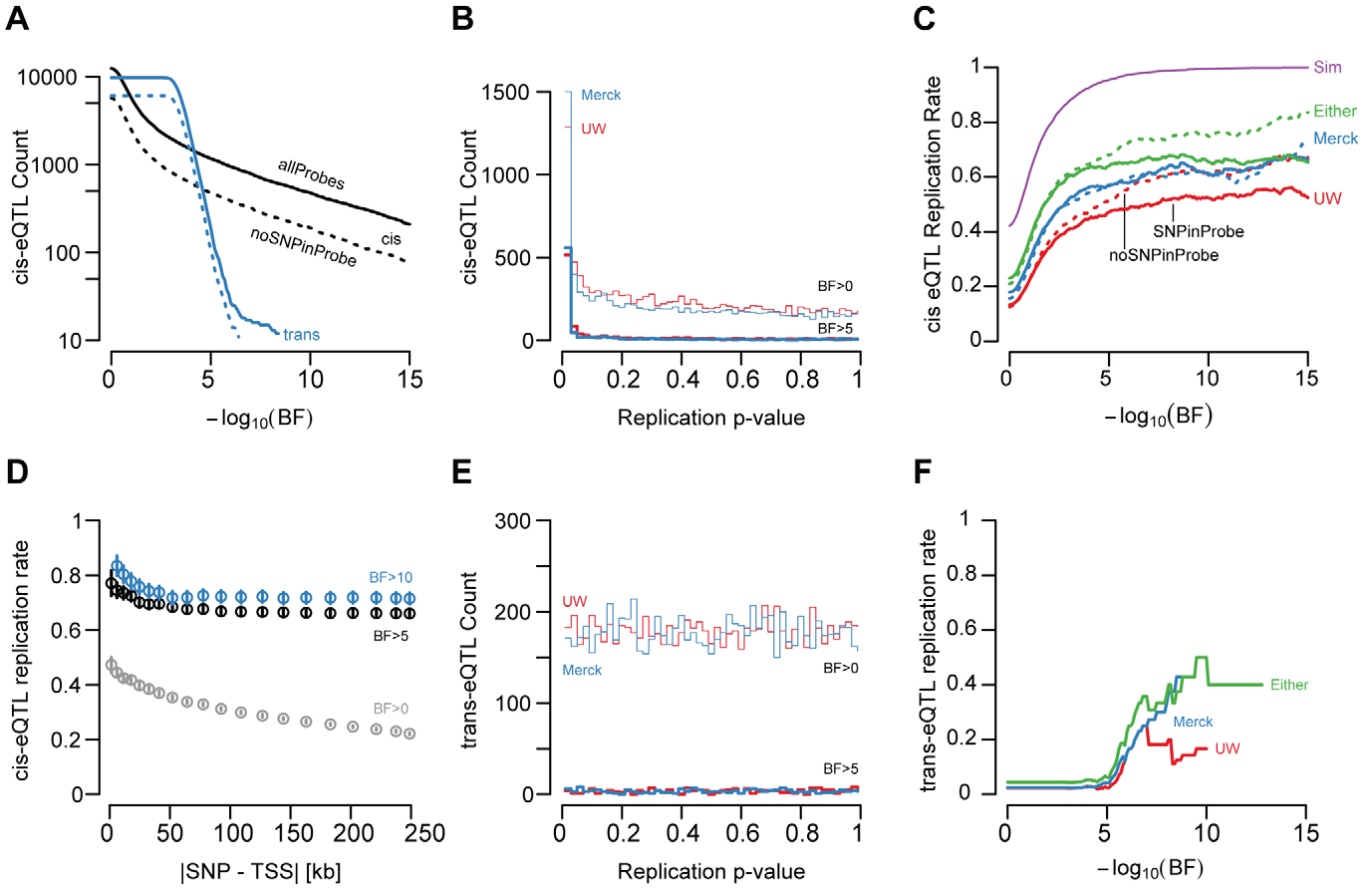
eQTL replication. (A) Number of gene expression traits (y-axis, log10 scaled) with best associated cis-eQTLs (black) and trans-eQTLs (blue) as a function of BF (x-axis). Counts at each threshold are plotted separately for all probes (solid) and for probes without known polymorphisms (dashed). (B) cis-eQTL associations were tested in two replication sample sets, UW and Merck. Replication sample linear regression t-test p-values were binned (x-axis) and the number of genes per bin (y-axis) is displayed separately for the UW (red) and Merck (blue) replication sets. Data are plotted for all eQTLs (thin lines) and for significant eQTLs (heavy lines). (C) cis-eQTL replication rate (y-axis) is depicted as a function of UC BF (x-axis) for UW (red), Merck (blue), either replication study (green), and for the n = 60 resampled data (purple). Replication rates are plotted separately for probes overlapping known polymorphisms (solid lines) and for probes not overlapping an annotated polymorphism (dashed). (D) Cis-eQTL replication rate (y-axis) plotted as a function of quantile binned SNP to TSS distance. Per bin mean (points) and standard errors (lines) are plotted separately for associations with UC BF>0 (grey) with UC BF>5 (black), and with UC BF>10 (blue). (E) Trans-eQTL replication p-values were binned (x-axis) and the number of genes per bin (y-axis) is displayed separately for the UW (red) and Merck (blue) replication sets. Data are plotted for all eQTLs (thin lines) and for significant eQTLs (heavy lines). (F) trans-eQTL replication rate (y-axis) is depicted as a function of UC BF (x-axis) for UW (red), Merck (blue), either replication study (green), and for the resampled data (purple).

After adjusting for age, sex, ancestry, and unmeasured confounding variables (quantified by surrogate variable analyses, see Methods and [20]), we found 1,787 gene models with significant cis-linked genetic effects on expression levels (UC log_10_ Bayes Factor (BF)>5; SNP to TSS distance <250 kb; Figure 2A, Figure 3A, Table S1). The distribution of t-test p-values in the replication sets, adjusted for the same covariates, for the UC best associated gene-SNP pairs were significantly enriched for small values (Figure 3B), indicating that a large fraction of cis-eQTLs are reproducible in independent sample collections. As with demographic effects, we defined replication as a p-value<0.05 and a concordant allele effect direction (Figure 3C). While the significance of association in the discovery cohort has a large effect on replication probability, the relationship between significance and replication was effectively binary (Figure 3C). Cis-eQTLs with BFs>5 were much more likely to replicate than those with BFs<5 (chi square p-value<2×10^−16^). However, among genes with BFs>5, replication probability was only weakly dependent upon BF (Figure 3C; logistic regression chi-squared p-value = 0.00319).

We found that 49.1% and 57.6% of significant cis-eQTLs (UC BF>5) replicated in the UW and Merck studies, respectively (i.e., p-value<0.05 and concordant effect directions; Figure 2A–2B, Figure 3C). The lower observed replication rate for the UW study is partially attributable to the smaller sample size (60 vs 266), but may also reflect platform-dependencies. 66.7% of significant cis-eQTL associations replicated in at least one of the two replication cohorts, while 47.6% replicated in both cohorts. Cis-eQTLs that replicated in one replication study were significantly more likely to replicate in the second replication study than expected by chance (chi-squared p-value<2×10^−16^) and twice-replicated eQTLs had larger effect sizes than eQTLs that replicate in only one study (Wilcoxon rank-sum test p-value<2×10^−16^; Figure S5, examples of non-replicated cis-eQTLs displayed in Figure S7).

### Sample size, statistical power, and winner's curse

Given differences in sample sizes among these studies, we sought to define a baseline replication rate against which to compare the observed levels of reproducibility. We therefore conducted a re-sampling experiment in which, for each gene expression trait, 100 sets of 60 sex and age (+/−3 years) matched samples were selected at random and used to define replication (*i.e.* concordant effect direction and *p*<0.05). We found that simulated replication rates increase dramatically near a BF of 5 (95.5% replication at BF>5; Figure 3C) and are effectively 100% at higher thresholds. These observations suggest that power differential among the studies cannot alone explain the observed rates of replication, as there are many genes with effect sizes in one study that should be readily detected in both (let alone either) replication panels. This is further supported by the observation that concordance alone (*i.e.* no p-value threshold) yielded similar levels of reproducibility, as did direct comparisons of allelic coefficients (Spearman's rho of 0.663 and 0.681 for UC–UW and UC-Merck comparisons, respectively; Figure S6).

We next sought to evaluate whether ‘winner's curse’ [33], [34] was deflating replication rates. Therefore, we extracted simulations in which the estimated coefficients randomly decreased and found that simulated replication remained >90% at BF>5 and near 100% at higher BF even when the effect size declined substantially (*e.g.* 30% drop in regression coefficient; Figure S9). Effect sizes would need to be over-estimated by 2-fold or greater across the entire set of eQTLs with UC BF>5 to result in the observed rates of replication. Furthermore, two lines of reasoning suggest winner's curse is not a major contributor to the observed rates of non-replication. First, we note that bias resulting from winner's curse should be progressively less pronounced as the true effect size increases, which in turn will correlate with significance estimates in the discovery panel [34]. However, replication rate was essentially flat even at extremely stringent thresholds (Figure 3C). Additionally, the resampling experiments demonstrated that, in direct contrast with a winner's curse prediction, effect sizes would need to be increasingly more severely over-estimated at higher thresholds (3-fold or more) to result in the observed rates of replication (Figure S8). Second, the definition of replication (concordance and p-value<0.05) is relatively loose when applied to eQTLs with a BF>5 (typical linear regression p-values<5×10^−8^) and should accommodate substantial drops in effect sizes for both replication panels but especially for the larger Merck dataset. This is further supported by the observation that concordance alone yielded similar rates of replication (Figure S6). We conclude that statistical power and winner's curse cannot explain the observed rates of non-replication for eQTLs with BF>5.

### On reproducibility failures due to hybridization artifacts

One possible explanation for non-replication is that SNPs within sequences targeted by expression probes may change hybridization efficiency in an allele-specific manner; if that SNP is also correlated with a genotyped variant, false positive eQTLs may result [35]. While 45.3% and 37.2% of Agilent and Illumina probes overlap with a polymorphism found in dbSNP131 or the one thousand genomes project (2010.08.04 release), the frequency distribution of polymorphisms in and around probe sequences differs markedly between the Agilent (UC) and Illumina (UW) platforms (Figure S9); Illumina expression probes have clearly been designed to avoid common polymorphisms.

The presence of SNPs in expression probes had a larger effect on reproducibility at extremely high thresholds (Figure 3C). For example, the replication rate for cis-eQTLs with BF>5 is not significantly affected by the presence of SNPs in probes (p-value = 0.189); however, replication rate for cis-eQTLs thresholded at BF>10 is significantly affected by probe SNPs (p-value = 0.0354; 65.6% with, 74.9% without SNP) and replication rate is significantly associated with an interaction between probe SNPs and eQTL significance (logistic regression BF-SNP interaction p-value = 0.0224). These results suggest the proportion of non-reproducible cis-eQTLs increases with eQTL significance such that, for eQTLs with BF>10, ∼27% of the non-replication rate can be explained by the presence of hybridization artifacts caused by known polymorphisms. To investigate the potential confounding role of unannotated polymorphisms in eQTL ascertainment, we re-sequenced 15 expression probes for genes that had large discrepancies in correlation measurements between the UW and UC studies that did not overlap a known SNP (9 probes with strong UW correlation but low UC correlation, 6 of the converse; Table S2). We found that none of these 15 probes harbored SNPs in the 60 UW liver samples or a panel of 35 CEU HapMap samples. Collectively, our data suggest future array designs/eQTL studies would benefit from more aggressive avoidance of known SNPs, but current SNP annotations are sufficiently comprehensive that unknown variants are of little concern to eQTL analyses.

### Surrogate variable analysis dramatically improves eQTL reliability

**Figure 4:**
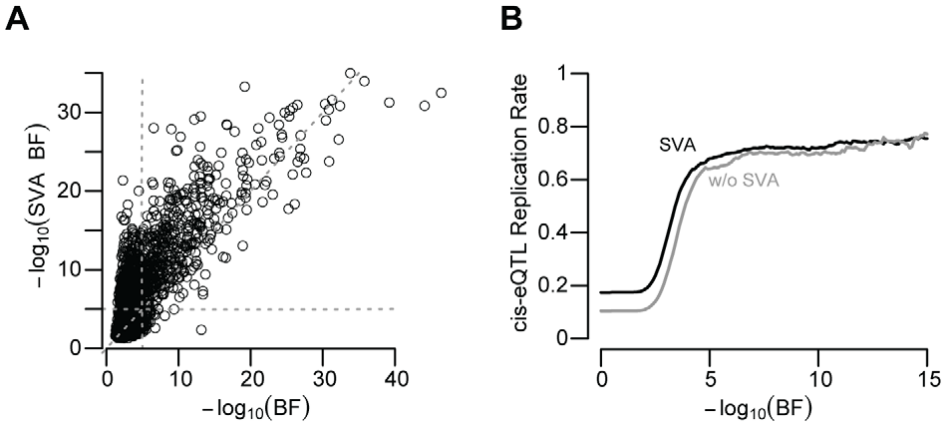
SVA improves eQTL reproducibility. (A) Surrogate variable regression produces more significant associations. Each point represents the BF for each UC gene expression trait and its best associated SNP. Data are plotted for associations tested after surrogate variable regression (y-axis) and unadjusted for surrogate variables (x-axis). Note that most points fall above the diagonal, indicating increased eQTL significance after surrogate variable correction. (B) Cis-eQTL replication rate (y-axis; UC vs UW or Merck) as a function of UC BF threshold. SVA adjusted associations depicted in black, unadjusted in grey.

We next quantified the role of several additional factors that may generate spurious associations. Most strikingly, failure to control for unknown or unmeasured confounding variables by surrogate variable analysis (SVA) produced a large decrease in the number of significant (BF>5) cis-eQTL signals (1,787 vs. 873; Figure 4A; McNemar's chi-squared test p-value<2×10^−16^), similar to a recent study of gene expression within twins [23]. Not only did SVA produce a larger number of significant cis-eQTL associations, but these associations were also significantly more likely to replicate (McNemar's Chi-squared test p-value≪2×10^−16^; Figure 4B). While it has been shown that unknown or unquantified confounders can lead to unreliable genetic predictions [19], [36], [2], our data show that such factors, if unaccounted for, dramatically decrease the number of eQTL signals and their reproducibility across multiple independent collections of primary human tissues.

### Other factors influencing reproducibility

Several additional aspects of the gene expression measurements correlated with cis-eQTL replication rate. Cis-eQTL replication rate was significantly associated with mean gene expression level and, independently, inter-individual expression coefficient of variation (Figure S10; multivariate logistic regression chi-squared p-value = 3.44×10^−3^ and 1.41×10^−4^, respectively); more highly expressed and highly variable genes were more likely to replicate. Further, we found that expression variance unexplained by age, sex, race, and surrogate variables was negatively correlated with expression level (Spearman's rho = −0.302, p-value<2×10^−16^). These data suggest greater measurement accuracy at higher expression levels that leads to more robust eQTL identification.

We also found that the best associated SNP for each gene expression trait was frequently immediately upstream or downstream from the transcription start site (TSS) (Figure 2C, [37]). Replication rate of significant cis-eQTLs was associated with absolute SNP to TSS distance (logistic regression chi-squared p-value = 5.35×10^−3^). 74.5% of cis-eQTLs within 5 kb of the TSS replicated, compared with only 60.6% located more than 100 kb from the TSS. Thus while distal regulatory elements are clearly important for human gene expression regulation, robustly quantifiable, segregating expression polymorphism was more likely to be found in SNPs very close to the TSS of genes.

Interestingly, significant cis-eQTLs were no more likely to replicate when analyses were restricted to probes targeting the same exon (chi-squared p-value = 0.759), demonstrating that most non-replicating eQTLs (in our study design) can not be accounted for by differential splicing or isoform usage. Similarly, replication was not improved when analyses were restricted to gene expression measurements derived from more than one expression probe (chi-squared p-value = 0.919). Additionally, the minor allele frequency of the associated SNP did not have a significant effect on replication rate (logistic regression chi-squared p-value = 0.600; Figure S10), and eQTLs at imputed SNPs replicated at similar rates to directly genotyped SNPs (logistic regression chi-squared p-value = 0.574; Figure S10). Uncertainty at imputed SNPs does not appear to have a significant effect on cis-eQTL replication rate, as the ratio of observed to expected genotype variance was not associated with replication rate in any of the three sample sets (logistic regression chi-squared p-values all >0.152; Figure S12).

Examination of the interplay of the factors influencing eQTL replication revealed several interesting trends. As mentioned above, replication probability was significantly associated with SNP to TSS distance, but this association decreases with increasing cis-eQTL significance (distance×BF interaction logistic regression p-value = 3.98×10^−5^). Thus, location information can help to differentiate real from false positive correlations of modest effect, but is less important for very strong correlations. We constructed stepwise multivariate logistic regression models, restricted to associations with BF>5, and confirmed that BF (logistic regression chi-squared p-value = 7.32×10^−3^), SNP to TSS distance (p-value = 2.33×10^−3^), gene expression (p-value = 0.0230), gene expression CV (p-value = 1.33×10^−4^), and probe SNP×BF interaction (p-value = 0.0207) all have significant effects on the cis-eQTL replication rate. In contrast, SNP minor allele frequency, SNP type (imputed or direct), and genotype variance do not substantially influence replication rate (p-values>0.5).

### Trans-eQTLs

We also conducted genome-wide scans for associations between gene expression traits and unlinked SNPs. Such trans-eQTLs may represent regulatory interactions between transcription factors, signaling molecules, or chromatin regulators and their target genes. After adjusting for demographic variables as above, we found 353 gene expression traits with significant (BF>5) trans-linked genetic effects. The replication behavior of trans-eQTLs was markedly different from cis-eQTLs (compare Figure 3B, 3C with Figure 3E, 3F). First, the distribution of t-test p-values derived from the UW replication set, for each best associated gene-SNP pair identified in the UC set, was effectively uniform (Figure 3E). Second, in contrast to cis-effects, which rapidly approach an asymptotic replication rate at BF 5, trans-eQTLs almost completely failed to replicate (6.14%; Figure 3F) at a BF threshold of 5. At greater significance thresholds, trans effects did replicate more frequently (e.g., at BF> = 9.5, 50.0% replicate), however, these rates never approached those observed for cis-eQTLs. It is plausible that surrogate variable correction may mask true ‘master’ regulator effects, but as for cis-effects we identified more trans-eQTLs with surrogate variable correction than without and these associations were more likely to replicate (data not shown). While it is perhaps surprising that even extremely significant trans effects frequently fail to replicate, we note that this behavior is, to a certain extent, to be expected [27].

### Fine-mapping and functional characterization

As the eQTLs we identified are associations between effectively anonymous SNPs and expression of a nearby gene, we were also interested in fine-mapping the associations, ideally to a causal variant (expression quantitative-trait-nucleotide or eQTN) or haplotype. We therefore re-sequenced the promoter and 3′UTR sequences for 18 genes with strong cis-eQTLs within the 60 UW livers (Table S3). Thirteen of these genes harbored a common SNP or indel within the proximal promoter or 3′UTR that correlated strongly (p-value<1×10^−8^) with the expression level of that gene, while 17 of 18 harbored a variant with at least a modest correlation (p-value<0.001). Of these 17 genes, the most strongly correlated SNP was within the 3′UTR for 11 genes and within the promoter region for 6 genes. Moreover, 10 of the 17 best SNPs were not within HapMap, indicating that a majority of the most strongly associated promoter/3′UTR variants were neither genotyped directly nor imputed and therefore not detectable in the original eQTL analysis.

**Figure 5:**
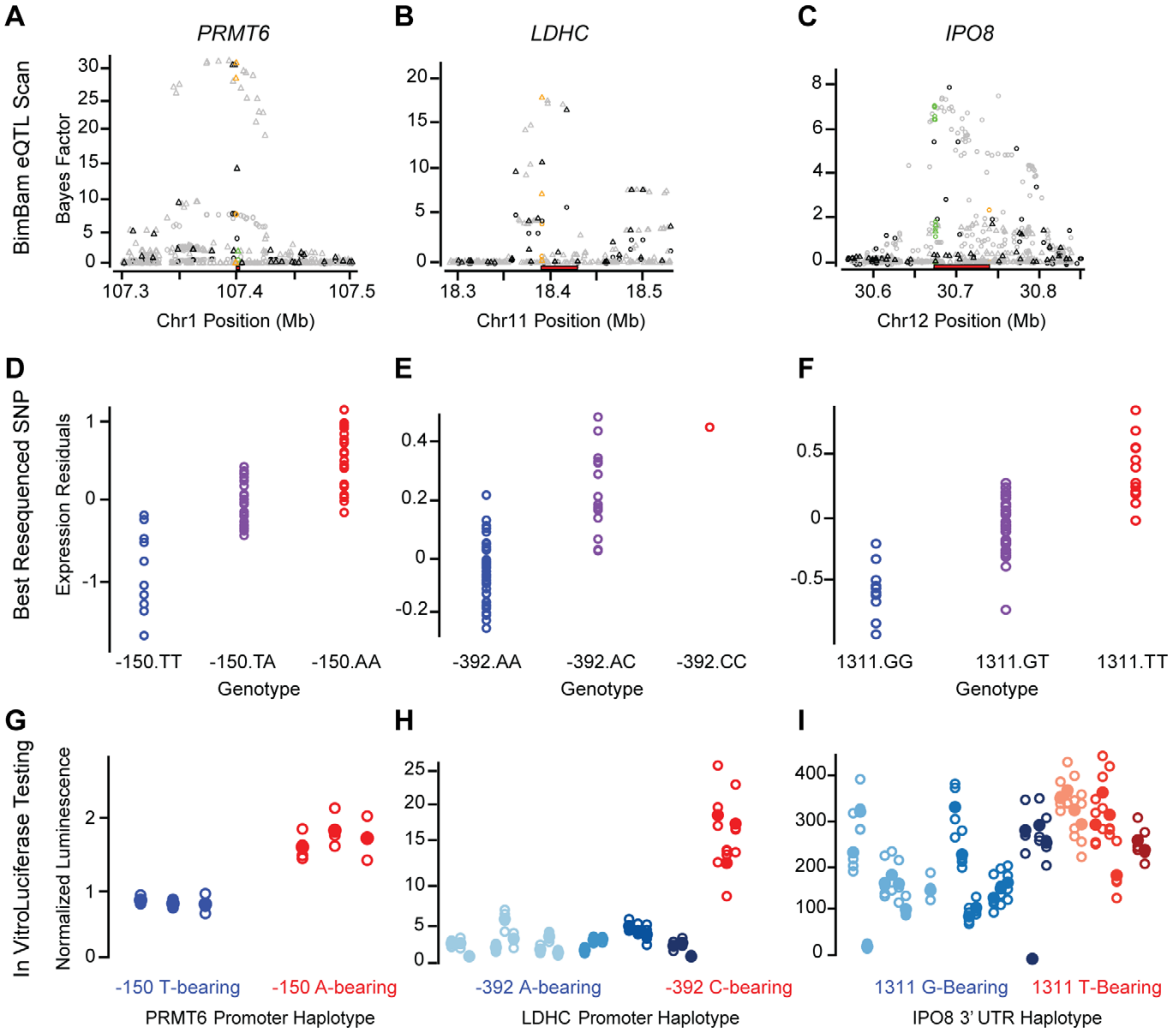
Fine-mapping functional results. Functional fine-mapping results for three genes, presented in columns: *PRMT6* (A, D, G), *LDHC* (B, E, H), and *IPO8* (C, F, I). (A–C) Cis-eQTL scan results are plotted across each gene region with chromosomal coordinates on the x-axis BF on the y-axis. Genotyped SNPs are plotted in black, imputed SNPs in grey, promoter SNPs in green, and 3′UTR SNPs in orange. The extent of each gene is plotted in a red rectangle near the X-axis. Association data from the UC study plotted as triangles and from the UW study as circles. (D–F) Putative eQTNs identified by promoter and 3′UTR resequencing. Microarray expression measurements (residuals after regression against covariates, y-axis) for each sample, plotted by genotype at the best-associated (ranked by p-value) SNP within the resequenced regions. Colors of the points correspond to the luciferase data. (G–I) Luciferase reporter results. Normalized luciferase measurements (y-axis) are plotted for each tested clone corresponding to a given haplotype (indicated by color). Blue and red coloring corresponds to the identity of each haplotype at the most strongly correlated individual SNP (same as in the middle row), while varying shades of red and blue differentiate haplotypes that differ at other SNPs. Vectors for each haplotype were prepared multiple independent times (data for each mini-prep are organized into a single column) and each mini-prep was transfected and measured four times (each open circle indicates one of these 4 measurements). The mean luminescence for each mini-prep is shown as a solid circle.

We subsequently sought experimental support for the functional nature of the most strongly associated SNPs. Therefore, for 14 genes, we cloned (and sequence-verified) common haplotypes existing in the UW liver samples into a customized luciferase reporter vector, and tested the function of each haplotype using high-throughput, transient transfection reporter assays (Table S3; 9 of 14 underlying cis-eQTLs replicated in the UC or Merck samples). For each haplotype, multiple independent vector (mode of 3) preparations were made, and for each plasmid preparation 4 transfection replicates were performed (mode of 12 measurements per haplotype). We analyzed the resulting data using a random-effects model that accounted for both variation in transfection replicates and variation in vector preparations. Our results underscore the need to perform multiple independent DNA preparations to reliably infer sequence-specific functional effects with this system (Figure 5 and data not shown).

We identified three regions where the haplotype sequence had a significant (p-value<0.001) effect on reporter activity (luminescence) in the same allelic direction as the expression measurements, including two promoters and one 3′UTR region (Figure 5 and Figure S12). No significant but discordant effects were observed. Variants near *PRMT6*, which encodes a protein-arginine methyltransferase and has been associated with HIV infection progression [38], scored highly in both the UW and UC eQTL analysis (Figure 5A). Resequencing of the *PRMT6* promoter yielded two common haplotypes defined by two perfectly correlated SNPs located 406 and 150 bp upstream from the TSS. The minor haplotype (40% frequency) correlated with a strong additive decrease in *PRMT6* liver expression (t-test p-value = 6.4×10^−14^ for UW), and relative to the major haplotype, we found a concomitant decrease in luminescence for reporter constructs harboring the minor haplotype (p-value = 0.0002). A similar result was obtained for promoter haplotypes of the *LDHC* (lactate dehydrogenase C) gene in which six common variants defined 7 common haplotypes, five of which were successfully cloned and tested. The strongest expression correlation was observed for a SNP located 392 bp upstream of the TSS (15% MAF), and the luciferase data strongly support the functional effect of this variant (p-value = 8.7×10^−9^; Figure 5B).

Finally, we identified a significant haplotype-specific effect within the 3′UTR for *IPO8* (importin 8), a protein that interacts with Argonaute proteins to direct miRNA mediated gene expression regulation [39]. There were nine common 3′UTR haplotypes defined by 13 variants for *IPO8*. The two haplotype groups defined by the most strongly expression-associated SNPs (two perfectly correlated variants at positions 1147 and 1195 relative to the 3′UTR start) have significantly different (p-value = 9.5×10^−4^) functional effects. However, unlike *LDHC*, there remained a substantial amount of variance within the haplotypes defined by alleles at these two SNPs, suggesting other variants may also have a functional role. Alternatively, the data gathered from 3′UTRs were generally noisier than that for promoters (Figure 5 and data not shown), and may not be as sensitive for identifying sequence-specific 3′UTR effects. Due to the increased noise, we repeated the analysis and performed new clone preparations and transfections for a subset of the *IPO8* haplotypes. The replicate data also show a significant (p-value = 0.007) difference, in the same direction, between haplotypes defined by their 1147 (or 1195) allele (Figure S12).

## Discussion

Genetic analyses of gene expression have great potential to facilitate insights into the genetic basis of complex traits. However, the utility of these data are limited by the extent to which the discovered associations correspond to legitimate, reproducible associations. Our estimates of 49% (UC vs. UW), 57% (UC vs. Merck), and 67% (UC vs. either) cis-eQTL reproducibility are substantially lower than two recent reports between two mouse crosses (76%, [27]), two independent sets of lymphoblastoid cell lines (83%, [25]), and two sets of primary human skin (>99%, [26]). Several non-exclusive possibilities likely contribute to these discrepancies. First, different discovery methodologies and replication criteria were employed in each study. Second, our studies were performed on different expression platforms (Agilent and Illumina), which reduces the influence of reproducible platform-specific errors but may result in missing splice-variant-specific eQTLs [40], [41], [10] as array manufacturers often target different exons in a given gene. However, this is likely to have a limited effect, as we found that the replication rate was not significantly different for genes assessed by probes within the same exon (Figure S10). Third, we compared three independent collections of primary human tissues (see Methods), not transformed cell lines or mouse tissues, and, despite the interpretive advantages associated with the former, our replication rate estimate is possibly downwardly biased by cell type heterogeneity. Finally, other systematic differences between studies, including protocols for sample collection and storage, clinical interventions taken by patients prior to death and autopsy, causes of death, life histories, *etc.*, may contribute to non-reproducibility. This hypothesis is supported by the observation that drug exposures and other clinical covariates, for which data limitations prevent comprehensive analysis, have substantial effects on gene expression; for example, we found that drug metabolism genes were significantly up-regulated in barbiturate-exposed *vs* non-exposed livers (data not shown). The striking difference in reproducibility between the results reported here and a recent report quantifying the overlap of human skin eQTLs [26], suggests that the degree of functional tissue heterogeneity may vary substantially across tissues.

An important caveat is that these estimates of reproducibility are less meaningful for sequence-based studies of gene expression, which offer advantages in dynamic range and measurement accuracy [9], [10]; sequencing is also largely immune to the SNP-in-probe effect that significantly inflates false positives in our data (Figure 3C). However, the observation that age, sex, race, drug exposures, clinical covariates, and other global factors have such strong influences on expression (e.g., this study and [36], [2]) coupled with observations in other studies and different tissues that factors like cause of death are relevant [42], suggests that much of the non-reproducibility is in fact driven by systematic differences in tissue source. Such differences will likely be important to all studies of primary tissue samples, whether assayed by arrays or by sequencing. The reproducibility of future results would benefit from analysis of samples from multiple centers with as much clinical information as possible. Furthermore, our results confirm previous observations that the effects of unknown, unmeasured, or unquantified covariates can confound genetic effects with structured error sources [19], [36], [2], and that controlling for these hidden confounders substantially boosts the rate of eQTL discovery [23]. Importantly, we demonstrated that not only are more eQTLs detected but that their reproducibility in independent collections of primary human tissue was also significantly higher.

Finally, through resequencing and a widely used *in vitro* assay system [43], we found that of 14 tested genes, two genes harbored functional eQTNs in the proximal promoter and one gene harbored functional eQTNs in the 3′UTR. The success rate of 3 in 14 suggests that a substantial number of eQTLs, and by extension any complex traits that they may influence, can be functionally isolated using the scalable assay system that we employed or potentially higher-throughput assays [44]. We note that some truly functional variants will not be detectable in these assays, either from being tested out of their genomic context or having effect sizes below the limit of detection afforded by the number of replicates used (*e.g.* [45]), and that the actual fraction of eQTLs with promoter or 3′UTR functional variation may be substantially higher. Considering that replication was significantly greater for eQTLs near the ends of genes relative to those further away (Figure 3D), our functional analysis also strongly supports the use of SNP to gene distance as an important contributor to the prior probability that any given SNP is a cis-eQTN [37]. While some eQTNs clearly reside outside these regions (e.g., [46]), the heavy enrichment for reproducible and experimentally tractable eQTNs, coupled with historical evidence supporting disease relevance [47], [48], suggests that the relatively small ‘promoter’- or ‘3′UTR’-ome target spaces may be valuable additions to exome-based disease resequencing efforts [49].

Given the ubiquitous importance of gene expression variance to phenotype, the known heritability of gene expression variance, and the great preponderance of non-coding functional elements in the genome [50], complex disease studies can benefit from eQTL analyses. Towards that end, we searched for correlations between replicated eQTL SNPs identified here and complex trait associated SNPs (R^2^>80%; Table 2, Table S4) in the NHGRI GWAS catalog (http://www.genome.gov/gwastudies/). These included several previously characterized mechanistic links to complex traits, such as *VKORC1* expression and warfarin drug response [51] and *SORT1* expression correlations with lipid levels and heart disease [13], both of which were originally identified using the UW liver panel described here. Additionally, these data support a relationship, which had previously been speculated but not shown to exist, between *NOD2* expression levels and leprosy risk [48], and novel hypotheses such as a link between expression of the uncharacterized *C2orf43* gene and prostate cancer risk [52].

**Table 2:** Selected overlap between liver eQTLs and GWAS SNPs.

Traits	GWAS SNPs	eQTL SNP	Chr	eQTL Gene	UC BF	Replication?
LDL cholesterol, Blood lipid traits, Coronary heart disease	rs12740374; rs660240; rs629301; rs646776; rs599839	rs12740374	1	*SORT1*	30.553	UW, Me
Body mass index	rs10838738	rs3817335	11	*MTCH2*	28.316	Me
Systemic lupus erythematosus	rs9271100	rs9271100	6	*HLA-DRB5*	23.186	UW
Height	rs10935120	rs11919350	3	*ANAPC13*	20.007	UW, Me
Activated partial thromboplastin time	rs2731672	rs2731672	5	*F12*	18.961	UW, Me
LDL cholesterol, Blood lipid traits, Coronary heart disease	rs12740374; rs660240; rs629301; rs646776; rs599839	rs12740374	1	*CELSR2*	18.657	UW, Me
Meningococcal disease	rs426736	rs1065489	1	*CFHR4*	17.785	Me
Warfarin maintenance dose	rs10871454; rs9923231	rs2303222	16	*VKORC1*	15.939	UW, Me
Cholesterol, total	rs10903129	rs1053438	1	*TMEM50A*	14.231	UW
Other erythrocyte phenotypes	rs2075671	rs11520986	7	*GIGYF1*	13.964	UW
Prostate cancer	rs13385191	rs13385191	2	*C2orf43*	13.87	UW, Me
Multiple sclerosis	rs703842	rs8181644	12	*TSFM*	12.711	UW, Me
QT interval	rs37062; rs7188697	rs4784051	16	*SETD6*	12.174	Me
Height	rs9487094	rs9487100	6	*SMPD2*	11.478	Me
Leprosy	rs9302752	rs9302752	16	*NOD2*	10.603	UW, Me
Chronic kidney disease	rs1933182	rs4970767	1	*ATXN7L2*	10.004	UW, Me
Hematological parameters	rs210135	rs210142	6	*BAK1*	9.517	UW, Me
Primary tooth development (number of teeth)	rs6504340	rs7207109	17	*HOXB2*	9.07	UW, Me
Multiple sclerosis; Height	rs1790100; rs11830103	rs1060105	12	*CDK2AP1*	8.968	UW, Me
Vertical cup-disc ratio; Esophageal cancer and gastric cancer	rs1547014; rs738722	rs1547014	22	*CHEK2*	8.827	Me
Height	rs10935120	rs9968172	3	*CEP63*	7.354	UW, Me
Type 1 diabetes	rs3825932	rs11638844	15	*CTSH*	7.25	UW, Me
Pulmonary function	rs10516526	rs10516525	4	*INTS12*	6.887	UW
Height	rs6060369; rs6060373; rs6088813	rs6141548	20	*UQCC*	6.824	UW, Me
Height	rs4886707	rs10220738	15	*MAN2C1*	6.441	Me
Cholesterol, total	rs10903129	rs12027135	1	*RHCE*	6.334	Me
Plasma coagulation factors	rs867186	rs867186	20	*PROCR*	6.077	Me
Bipolar disorder	rs11622475	rs11625697	14	*TDRD9*	5.742	UW, Me
Bone mineral density (spine)	rs2016266; rs10876432	rs6580942	12	*ESPL1*	5.689	Me
Mean corpuscular hemoglobin	rs11085824	rs11085825	19	*GCDH*	5.569	UW, Me
Conduct disorder (interaction)	rs2282301	rs12037177	1	*RIT1*	5.521	UW, Me
Factor VII	rs561241	rs7981123	13	*F7*	5.477	UW, Me

In summation, our data facilitate insights into the factors and experimental design criteria that affect eQTL reproducibility and may improve future eQTL studies, replicate many published but nonreplicated eQTLs (*e.g.* from [31]), support and extend eQTLs identified in other tissues like brain (*e.g. FAM119B* [53]), identify many novel reproducible liver eQTLs, show that promoters and 3′UTRs are enriched for experimentally accessible functional variation, and support or suggest numerous mechanistic links to biomedically important phenotypes. We believe that this study and others like it will be valuable to the robust discovery and fine-mapping of the genetic basis for complex human diseases.

## Methods

### Ethics statement

Research conducted in this study was performed on deceased, anonymous individuals and is therefore not considered to involve ‘human subjects.’ Samples were collected with approval of institutional review boards (IRBs) and the University of Chicago and University of Washington IRBs approved their use for the purpose of this study.

### Tissue procurement—UC

Livers were processed through Dr. Mary Relling's laboratory at St. Jude Children's Research Hospital, part of the Pharmacogenetics of Anticancer Agents Research (PAAR) Group, and were provided by the Liver Tissue Cell Distribution System funded by NIH Contract #N01-DK-7-0004/HHSN267200700004C and by the Cooperative Human Tissue Network. Samples were collected with approval of institutional review boards (IRBs) and the University of Chicago IRB has approved their use for the purpose of this study.

Analysis began with 240 normal (non-diseased) livers that were collected from unrelated donors of self-reported European and African descent. Most of the liver tissue samples come from donor livers that were not used for whole organ transplants, the remainder being from liver tissue which remains following a partial graft into a smaller recipient, usually a pediatric patient. As such, each liver is procured with the intent to transplant under the best possible conditions to maintain cell viability. Standardized procedures have been in place for handling, freezing and storage of the livers and their subcellular fractionation and enzyme characterization. Demographic information is summarized in Table 1.

### Tissue procurement—UW

The University of Washington IRB approved the collection of the liver tissues and their subsequent use for the purposes of this study. Samples of human liver were obtained from organ donors through the University of Washington Transplant Program and the Northwest Organ Procurement Agency. Consent for research was obtained in all cases. Standard procedures were employed for the handling, freezing and storage of the livers.

### Gene expression analysis—UC

Gene expression microarray experiments were conducted with biological replication in all samples. Sample processing order was randomized. For each sample, total RNA was extracted at least twice independently, from tissue homogenized in TRIzol reagent, followed by Qiagen RNAeasy cleanup (Qiagen). RNA quality was assessed by Bioanalyzer (minimum RIN = 7). cRNA was produced using the Agilent Low-Input Linear amplification and labeling kit.

Array hybridizations (Agilent-014850 4×44 k arrays, GPL4133) were performed at The University of Chicago, Argonne National Labs high throughput genome analysis core facility, according the manufacturers instructions. The Agilent FE software was used to extract feature intensities and to flag saturated, non-uniform, and outlier features. Probe intensity was adjusted by subtracting background intensity using the minimum method [54], [55] and quantile normalized between arrays [56]. Dixon's outlier test was used to remove 13 arrays (out of a total of 517) based on total number of flagged probes, intra-array variance, inter-array variance, biological replicate variance, and spike-in linearity [57].

Probes were grouped into probe sets by aligning first to RefSeq gene annotations and then aligning unmapped probes to the human reference genome (build 36). All probes with non-unique best alignments were excluded from further analysis. Multiprobe probesets were hierarchically clustered using one minus the pearson correlation coefficients as a distance matrix. Clusters were divided into groups by cutting clusters at a dendrogram height of 0.5 (roughly producing clusters with internal correlation coefficients >0.5). All downstream analyses were performed independently on each resulting cluster and all single probe probesets.

### Gene expression analysis—UW

Total RNA was extracted from 60 human liver tissue samples from the University of Washington School of Pharmacy Human Liver Bank as previously described [51], [13]. Genome wide expression analysis was performed using 750 ng of total RNA on the Illumina HumanRef-8 v.2 platform (GPL5060). All liver samples were analyzed with technical replicates that were randomized between processed batches of 24 arrays performed on different days. Raw signal intensity measurements from each sample were processed using the Illumina BeadStudio software v. 2.3.41 using the ‘average’ normalization function. Replicate data from each liver was averaged prior to statistical analysis. All samples and replicates passed quality-control measures.

### Gene expression analysis—Merck

Processed gene expression data from the published Merck liver eQTL study [31] were downloaded from GEO (GSE9588, GPL4371). Based on available sample metadata, 266 samples had (a) unambiguous sample ID, age and sex assignments (b) expression data, (c) genotype data, and (d) did not overlap with the UC study. Probes were grouped into RefSeq gene annotation probe sets based on the array manifest. Probesets were further clustered and split following the methodology used for the UC array set.

### Genotyping—UC

From the same liver samples received from the Liver Tissue Resource, DNA was obtained from 240 samples for genotyping. Genotyping was performed on the Illumina human 610 quad beadchip platform (GPL8887) at the Northwestern University Center for Genetic Medicine Genomics Core Facility according to the manufacturer's instructions. One sample was removed because it had a no call rate >10%. The initial marker set comprised 620,901 markers. 8,300 markers were removed because they showed significant deviation from Hardy-Weinberg equilibrium (HWE, Fischer's exact test, p<0.001). 29,705 SNPs were removed from the analysis because they had a no call rate in more than >10% of the samples. Hence, our final marker set is comprised of 583,073 SNPs. Identity by descent analysis, performed in Plink, revealed 14 pairs of duplicated samples. Erroneous, redundant sample collection was later confirmed by the tissue bank. Genotype and expression data for these samples were merged for all downstream analyses. The final sample set therefore consisted of 225 unique samples.

### Genotyping—UW

Genotyping was performed on each liver sample using the Illumina HumanHap550 (GPL6981) Beadchip platform. Genotyping calls were made using GenomeStudio. After raw genotyping data were loaded into the software, pre-defined cluster definitions were applied and genotype calls were determined. Clusters were checked for separation, deviation from HWE, and lack of variation (i.e., monomorphic). Poorly assigned clusters were modified manually and sites were re-called with corrected cluster definitions. All samples had call rates greater than 98%.

### Genotyping—Merck

Genotype data were generated as described [31].

### Sex confirmation

The sex of each sample was imputed by K-means clustering of Y-linked gene expression levels and X- and Y-linked genotypes. 3 UC samples, 0 UW samples, and 0 Merck samples had mismatched imputed and annotated sexes, and were therefore excluded from all analyses.

### Genotype imputation

For all three studies, care was taken to translate all genotypes to reference genome (b36) forward strand alleles, as subtle errors in genotype strand inference will downwardly bias replication rate estimates. Additional genotypes were imputed with Bimbam (v 0.99) [58], using HAPMAP release 27, build 36 unphased genotypes as reference panels. European American genotypes were imputed with a CEPH reference panel, while African American genotypes were imputed with a combined CEPH and YRI panel. Imputation was run with default Bimbam parameters, and mean imputed genotypes were recorded and used for all downstream analyses.

### Quantification of ancestry—UC

We performed a principal component analysis (PCA) based quantification of race using the African and European populations from the Human Genome Diversity panel as reference populations. The SNP set was trimmed using linkage disequilibrium (LD)-based SNP pruning, removing all SNPs for with high pairwise LD (R^2^>0.8), as in [59]. PCA was performed using smartpca, as implemented in EIGENSOFT [60]. Four samples were flagged as outliers and removed from all further analyses. As expected, the first principal component separated African from non-African individuals. We therefore used this loading vector as an estimated quantification of African ancestry for further analyses.

### Quantification of ancestry—UW

PCA was performed using the multi-dimensional scaling procedure implemented in PLINK v1.06 (http://pngu.mgh.harvard.edu/purcell/plink/) [61]. The vast majority of samples resided in a single cluster including all the individuals of self-reported European ancestry, with several moderately outlying samples corresponding to self-reported Hispanic and African ancestry. No samples were excluded from further analyses; the vectors determined for the first two principal components were used as ancestry control for all statistical analyses.

### Quantification of ancestry—Merck

All 266 samples included from the published Merck study were self-reported Caucasians. The SNP set was trimmed using linkage disequilibrium (LD)-based SNP pruning, removing all SNPs for with high pairwise LD (R^2^>0.8), as in [59]. PCA was performed using the multi-dimensoinal scaling procedure implemented in PLINK v1.07 (http://pngu.mgh.harvard.edu/purcell/plink/) [61]. No outliers were detected; the vectors determined for the first four principal components were used as ancestry control for all statistical analyses.

### Covariate modeling—UC

For each probeset, surrogate variable analysis (SVA) [20] was performed on the matrix of expression measurements, after controlling for the effects of hybridization protocol, age, sex, and a principal component analysis based quantification of genetic ancestry. For each probeset, we then constructed a linear mixed effects model *y ∼ m + P + A + C + R + I + W + SV_i..n_ + e*, where *y* is the log_2_ transformed probe intensity, *m* is the expected probe intensity, *P* is a factor controlling for the effect of subtle variations in hybridization protocol (e.g., the identity of the technician who performed the experiment), *A* is the effect of individual age, and *C* is the effect of individual sex, and *R* is the effect of genetic ancestry. *I* is the random effect of each individual, *W* is the random effect of the oligonucleotide probe, *SV_i..n_* represents the effects of a matrix of 55 surrogate variables, and *e* is the residual error. The model was fitted to each gene by residual maximum likelihood using the lmer function in the R package lme4 (v 0.999375-32) [62], [63]. Fixed effect p-values were estimated using the pvals.fnc function in the languageR package (v 1.0) [64]. The significance of covariate effects was assessed by estimating false discovery rates, using Storey's q-value method [65]. To further control for the effects of outliers and population stratification, prior to eQTL mapping, the distribution of estimated individual effects, for each gene expression trait, was normal quantile transformed, within populations.

### Covariate modeling—UW

SVA [20] was performed on the matrix of expression measurements, after controlling for the effects of age, sex, and a multidimensional scaling based quantification of genetic ancestry. For each probe, we constructed a linear model *y ∼ m + A + C + R + SV_i..n_ + e*, where *y* is the log_2_ transformed probe intensity, *m* is the expected probe intensity, *A* is the effect of individual age, and *C* is the effect of individual sex, and *R* is the effect of genetic ancestry, *SV_i..n_* represents the effects of a matrix of surrogate variables, and *e* is the residual error. Models were implemented with the lm function in R. The residuals from this regression were used as the phenotype values for all subsequent analyses.

### Covariate modeling—Merck

SVA [20] was performed on the matrix of expression measurements, after controlling for the effects of age, sex, and a principal component analysis based quantification of genetic ancestry; 54 significant surrogate variables were identified. For each probeset, we then constructed a linear model *y ∼ m + A + C + R + W + SV_i..n_ + e*, where *y* is the log_2_ transformed probe intensity, *m* is the expected probe intensity, *A* is the effect of individual age, and *C* is the effect of individual sex, and *R* is the effect of genetic ancestry, *W* is the effect of the oligonucleotide probe, *SV_i..n_* represents the effects of a matrix of surrogate variables, and *e* is the residual expression. Models were implemented with the lm function in R. The residuals from this regression were used as the phenotype values for all subsequent analyses.

### eQTL mapping

For each gene expression trait, residual expression variance was treated as a quantitative trait and tested for association with all markers genome-wide. Association testing was performed by Bayesian regression, as implemented in Bimbam (v 0.99), using mean imputed genotypes and default priors [28], [29]. Genotypes with minor allele frequencies less than 1% were excluded.

### Probe resequencing

For 15 probes that showed discrepant eQTL scores between the UC and UW analyses (*i.e.* BF>4 in one study and BF<4 in the other), we designed primers to capture the relevant expression array probe and amplified and Sanger-sequenced the resulting PCR products in each of the 60 UW liver samples and 35 CEU HapMap samples. SNPs were identified as previously described (http://pga.gs.washington.edu/) including both automated prediction and manual curation.

### Fine-mapping

We resequenced the promoter and 3′UTR regions within the 60 UW liver samples and 35 CEU HapMap samples for 18 genes that showed strong expression-SNP correlations within the UW data (selected before replication information was available). We used PCR amplification and Sanger-sequencing, identifying SNPs using both automated prediction and manual curation as previously described (http://pga.gs.washington.edu/). 3′UTRs were defined using the appropriate gene models, while promoters were defined as the 1 kb segment upstream of the annotated transcriptional start site. We subsequently defined haplotypes within each promoter and 3′UTR as previously described using Phase [58], and designated as common all haplotypes present in at least two samples.

Common haplotypes for each of 14 promoter and UTR regions were PCR-amplified and cloned into luciferase-reporter vectors. Promoter haplotypes were cloned immediately upstream of the luciferase reporter gene, while 3′UTRs were placed at the 3′ end of a luciferase gene whose expression is driven by the *RPL10* promoter that has strong constitutive activity (vector maps available from SwitchGear Genomics, http://switchgeargenomics.com/resources/vector-maps/). We then transfected each of these constructs into HEPG2 cells, a liver-derived cell line, and measured luminescence. Each haplotype was tested using multiple (mode = 3) vector preparations and 4 technical transfection replicates measurements were obtained for each vector preparation (12 or more measurements for most haplotypes).

Transient transfection reporter assays were all performed in 96-well format. Transfection complexes were formed by incubating 100 ng of each individual promoter construct with 0.3 µL of Fugene 6 transfection reagent and Opti-MEM media in a total volume of 5 µL and incubated for 30 min. Transfection complexes (5 uL) were added to 10,000 HepG2 cells in 96-well format that had been seeded 24 h prior to transfection in a white tissue-culture treated plate.

After seeding and transfection, cells were incubated for 48 h before freezing at −80 degrees overnight. To read luminescent activity, plates were thawed for 45 min at room temperature. Then 100 µL of Steady-Glo reagent (Promega #E2520) was added and incubated for 30 min at room temperature. Then luminescence was read for 2 s per well on a 384-well compatible plate luminometer (Molecular Devices LMax384).

To identify significant *in vitro* effects of haplotype on luminescence, we employed a mixed-effects model using the lmer package [63] within R [62], grouping the replicate luminescence measurements by mini-prep identifier (treating the mini-prep as a random effect). The haplotype identifier has a significant effect on luminescence at p-value<0.001 for each of the three reported associations between haplotype sequence and luminescence measurement. No additional correlations were significant at this threshold.

## Supporting Information

Figure S1 UW best SNP analyses. (A) Number of gene traits (y-axis) with best associated cis-eQTLs in bins of increasing association significance (x-axis). (B) Distribution of distances from each gene's best associated SNP to its transcription start site (TSS). Negative and positive values denote SNPs 5′ and 3′ of TSS, respectively. (C) Distribution of linear regression SNP-expression association t-test p-values from the UC (red) and Merck (blue) sample sets for all genes and their most associated cis-SNP in the UW study. Gene counts (y-axis) are plotted per p-value bin (x-axis). Data are plotted for all eQTLs (thin lines) and for significant eQTLs (heavy lines). (D) Between study cis-eQTL effect replication rate (y-axis) plotted as a function of UW cis-eQTL significance threshold (x-axis). UW vs. UC (red), UW vs. Merck (blue), and UW vs. either (green) replication rates are plotted separately . (E) cis-eQTL replication rate (y-axis) as a function of distance from the best associated SNP to the gene TSS (x-axis). Data are plotted separately for eQTLs with BFs>0 (grey) and BFs>5 (black). eQTLs were binned in 2.5% quantiles; mean (circle) and standard error of the mean (bar) are plotted for each bin. (F) cis-eQTL replication rate (y-axis) as a function of the linear model minor allele count fixed effect coefficient (x-axis). Data are plotted as in E. (G) Between study cis-eQTL effect correlation coefficient (y-axis) plotted as a function of UW cis-eQTL significance threshold (x-axis). UW vs. UC (red), UW vs. Merck (blue).
(EPS)

Figure S2 Mean square (MS) distributions for each factor across all three studies. For the UC (red), Merck (blue), and UW (green) datasets, the average MS value for all genes is plotted (open circles, y-axis) for each indicated covariate (x-axis) in a model that includes all covariates, Error bars are drawn from one standard error (s.e.m) above to one standard error below the mean.
(EPS)

Figure S3 Examples of age- and sex-associated genes. Surrogate variable adjusted per-probe, per-sample residual expression data are depicted for six genes. Three genes (top row: *CD40*, *FGF2*, *HDAC1*) are significantly associated with sex and three genes (bottom row: *PPARA*, *HRAS*, *TMEM22*) are significantly associated with age. Each point represents the expression level of a single individual, as measured by a single gene expression probe. Data from males are plotted in black, females in red. Linear regression coefficient t-test p-values are provided.
(EPS)

Figure S4 Correlation of covariate effects. (A,B) Correlation coefficient (y-axis) for sex (A) and age (B) regression coefficients as a function of discovery sample association p-value (x-axis), plotted separately for UC-UW (red) and UC-Merck (blue) comparisons.
(EPS)

Figure S5 Comparison of singly and doubly replicating cis-eQTLs. (A) Effect size and (B) Bayes Factor distributions for singly and doubly replicated eQTLs.
(EPS)

Figure S6 Alternate replication metrics for eQTLs. (A) Correlation coefficient between linear model minor allele count fixed effect regression coefficients (y-axis) as a function of discovery sample cis-eQTL significance (x-axis). Correlations were calculated separately between UC and UW (red) and between UC and Merck (blue). Imputed cis-eQTL SNPs are plotted in dashed lines and directly genotyped SNPs are plotted in blue. (B) Concordance rates between linear model minor allele count fixed effect regression coefficients (y-axis) as a function of discovery sample cis-eQTL significance (x-axis). Concordance was calculated separately between UC and UW (red), between UC and Merck (blue), and between UC and either Merck or UW (green). Note that we adjusted the raw concordance rates to account for the fact that 50% of all false positives would replicate using this definition for a single replication panel and 75% would replicate in the ‘either’ category (50% of false positives using one replication panel, 50% of the remainder using the second). So, for example, a concordance rate of 80% between UC and a given replication panel results in a replication estimate of ∼60%, since we assume that the 20% of eQTLs that are discordant represent only half of all false positives. Similarly, a concordance rate of 90% using an ‘either’ standard also results in a replication estimate of 60%, since we assume that the 10% of effects that are discordant represent only a quarter of all false positives.
(EPS)

Figure S7 Examples of replicating and non-replicating cis-eQTLs. Residual gene expression (left y-axis) or raw gene expression measurements (right y-axis) plotted as a function of minor allele count (x-axis). Left column depicts UC data, center column Merck data, and right column UW data. Three UC cis-eQTLs that (A) replicate in UW but not Merck, (B) replicate in Merck but not UW, (C) that replicate in neither study, and (d) that replicate in both.
(TIF)

Figure S8 Simulation based estimates the relationship between effect size bias (winner's curse) and replication rate. (A) Age (+−3 years), sex, and race matched sets of 60 individuals were sampled from the UC data set and used to calculate a baseline replication rate. Replication rate (y-axis) is plotted as function of the ratio of full-dataset to resampled minor allele fixed effect coefficients (x-axis). Data are plotted separately for cis-eQTL sets that were thresholded at varying BF values. (B) The distribution of observed to resampled minor allele fixed effect coefficients were binned (x-axis) and the density of simulations per bin is plotted on the y-axis. As in (A), data are plotted separately for cis-eQTL sets that were thresholded at varying BF values.
(EPS)

Figure S9 Distribution of SNPs within and flanking Agilent, Illumina expression probes. Distribution of SNP counts (y-axis) at varying distances from the start coordinate of each expression probe (x-axis), depicted for both the Agilent (A) and Illumina (B) expression arrays. Black bar delineates the extent of the probe sequence. Note that Agilent and Illumina probes are 60 and 50 nucleotides long, respectively.
(EPS)

Figure S10 Expanded analysis of determinants of replication probability. (A) Between study cis-eQTL effect replication rate (y-axis) plotted as a function of UC cis-eQTL significance threshold (x-axis). Data are plotted separately for probes sets for which both the UC and UW expression array probes target the same exon (grey) and those for which they target different exons (black). Differences are not significant. (B–H) Replication rate between the UC and UW or Merck studies (y-axis) for all cis-eQTLs with BF>0 (grey), BF>5 (black), and BF>10 (blue) whose probes overlap a known polymorphism. (B) Cis-eQTLs are binned by the distance of the SNP from the 5′ end of the microarray expression probe (x-axis). Mean replication rate (points) and standard error of the mean (lines) are plotted per bin. (C) Cis-eQTLs are binned by the number of known polymorphisms overlapping the expression probe (x-axis).(D) Cis-eQTLs are binned by mean log_2_ gene expression level (x-axis). (E) Cis-eQTLs are binned by the coefficient of variation of log_2_ gene expression levels (x-axis). (F) Cis-eQTLs are binned by the linear model minor allele count fixed effect regression coefficient (x-axis), as estimated from the discovery samples. (G) Cis-eQTLs are binned by the mean residual linear model variance (x-axis), after adjusting for demographic and technical covariates. (H) Cis-eQTLs are binned by minor allele frequency (x-axis). (I) Between study cis-eQTL effect replication rate (y-axis) plotted as a function of UC cis-eQTL significance threshold (x-axis). UC vs. UW (red), UC vs. Merck (blue), and UC vs. either (green) replication rates are plotted separately . Replication rates are plotted separately for SNPs that were directly genotyped (dashed lines) and those that were imputed (solid lines). (J) UC-UW cis-eQTL effect replication rate (y-axis) plotted as a function of UC cis-eQTL significance threshold (x-axis). Replication rates are plotted separately for SNP pairs for which both SNPs were directly genotyped (red), both SNPs were imputed (green), and for which one SNP was imputed and one was directly genotyped (blue). (K) UC-Merck cis-eQTL effect replication rate (y-axis) plotted as a function of UC cis-eQTL significance threshold (x-axis). Replication rates are plotted separately for SNP pairs for which both SNPs were directly genotyped (red), both SNPs were imputed (green), and for which one SNP was imputed and one was directly genotyped (blue). (L) Replication rate between the UC and UW or Merck studies (y-axis) for all cis-eQTLs with BF>5. Cis-eQTLs are binned by minor allele frequency (x-axis) and plotted separately for imputed (orange) and directly genotyped (black) SNPs.
(EPS)

Figure S11 Imputation quality and replication. (A–C) Histograms depicting the number of imputed (red) and directly genotyped (blue) SNPs (y-axis) binned by the ratio of observed over expected genotype variance (x-axis). Expected genotyped variance calculated based on observed HAPMAP genotype frequencies. Data are plotted separately for UC (A), UW (B), and Merck (C) genotypes. (D) Replication rate between the UC and UW or Merck studies (y-axis) for cis-eQTLs with BF>0 (grey), BF>5 (black). Cis-eQTLs are binned by the UC ratio of observed to expected (based on CEU minor allele frequencies) genotype variance (x-axis). (E) Replication rate between the UC and UW (y-axis) for cis-eQTLs with BF>0 (grey), BF>5 (black). Cis-eQTLs are binned by the UW ratio of observed to expected genotype variance (x-axis). (F) Replication rate between the UC and Merck (y-axis) for cis-eQTLs with BF>0 (grey), BF>5 (black). Cis-eQTLs are binned by the Merck ratio of observed to expected genotype variance (x-axis).
(EPS)

Figure S12 Replication of *IPO8* 3′ UTR expression effect. Reporter construct clones from each 3′ UTR haplotype were prepared and transfected independently of the data presented in Figure 5. Data depicted as in Figure 5, bottom panel.
(TIF)

Table S1 All gene eQTLs. Each gene and all three studies, covariate effects, eQTL effects, linear model coefficients, Bayes Factors, SVA effects, and UC best-associated SNP annotation.
(BZ2)

Table S2 Expression probe re-sequencing.
(XLSX)

Table S3 Luciferase results table.
(XLSX)

Table S4 Extended overlap of GWAS associations and liver eQTLs, including all UC best-associated gene-SNP pairs regardless of BF or replication status.
(XLSX)
