# Supplementary figures and images for: Identification, Replication, and Functional Fine-Mapping of Expression Quantitative Trait Loci in Primary Human Liver Tissue
Source: PLoS Genet. 2011 May 26;7(5):e1002078. doi: 10.1371/journal.pgen.1002078 (PMC3102751; doi:10.1371/journal.pgen.1002078)

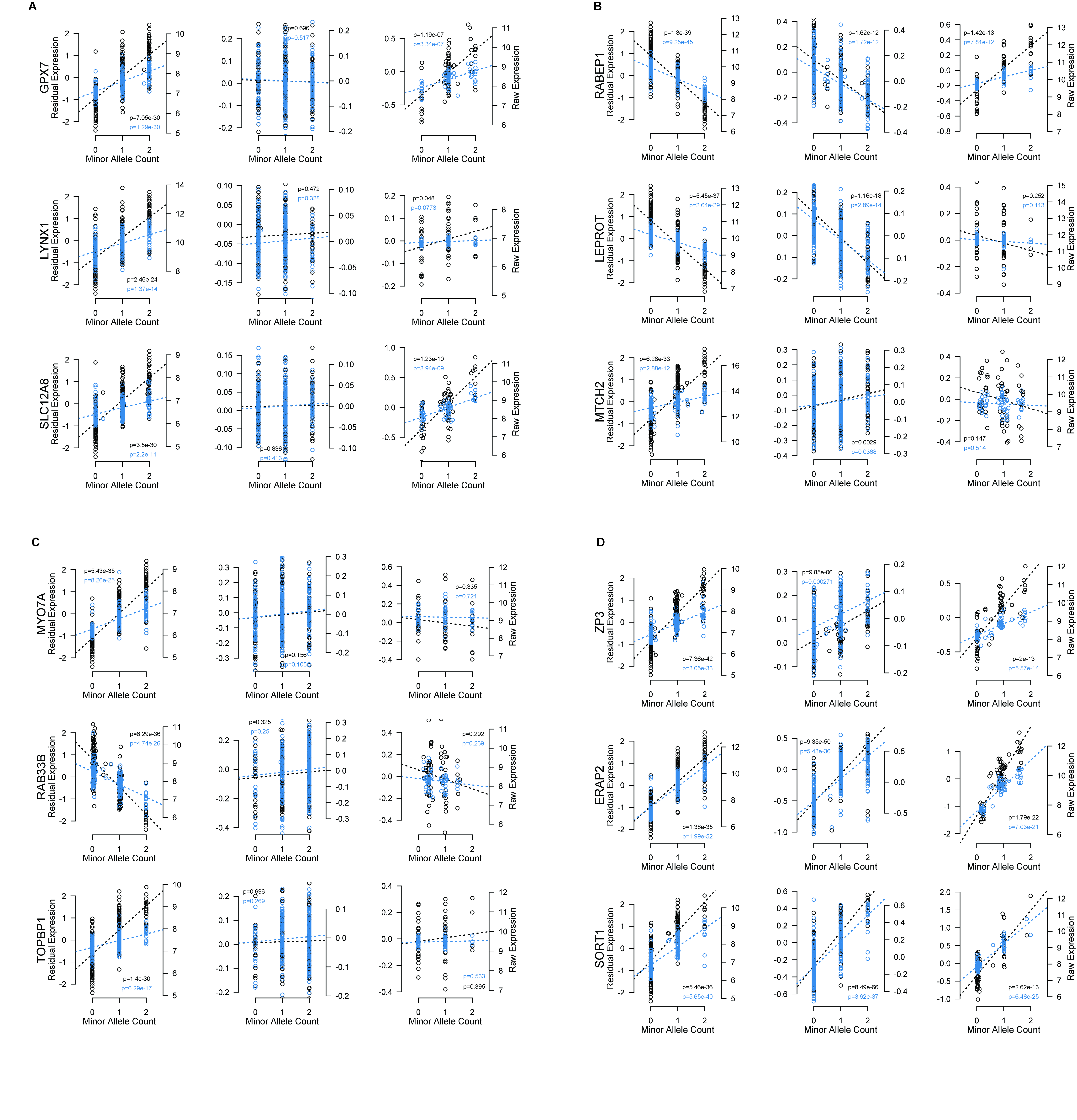

Supplement: Figure S7 — Examples of replicating and non-replicating cis-eQTLs. Residual gene expression (left y-axis) or raw gene expression measurements (right y-axis) plotted as a function of minor allele count (x-axis). Left column depicts UC data, center column Merck data, and right column UW data. Three UC cis-eQTLs that (A) replicate in UW but not Merck, (B) replicate in Merck but not UW, (C) that replicate in neither study, and (d) that replicate in both. (TIF) [file pgen.1002078.s007.tif]

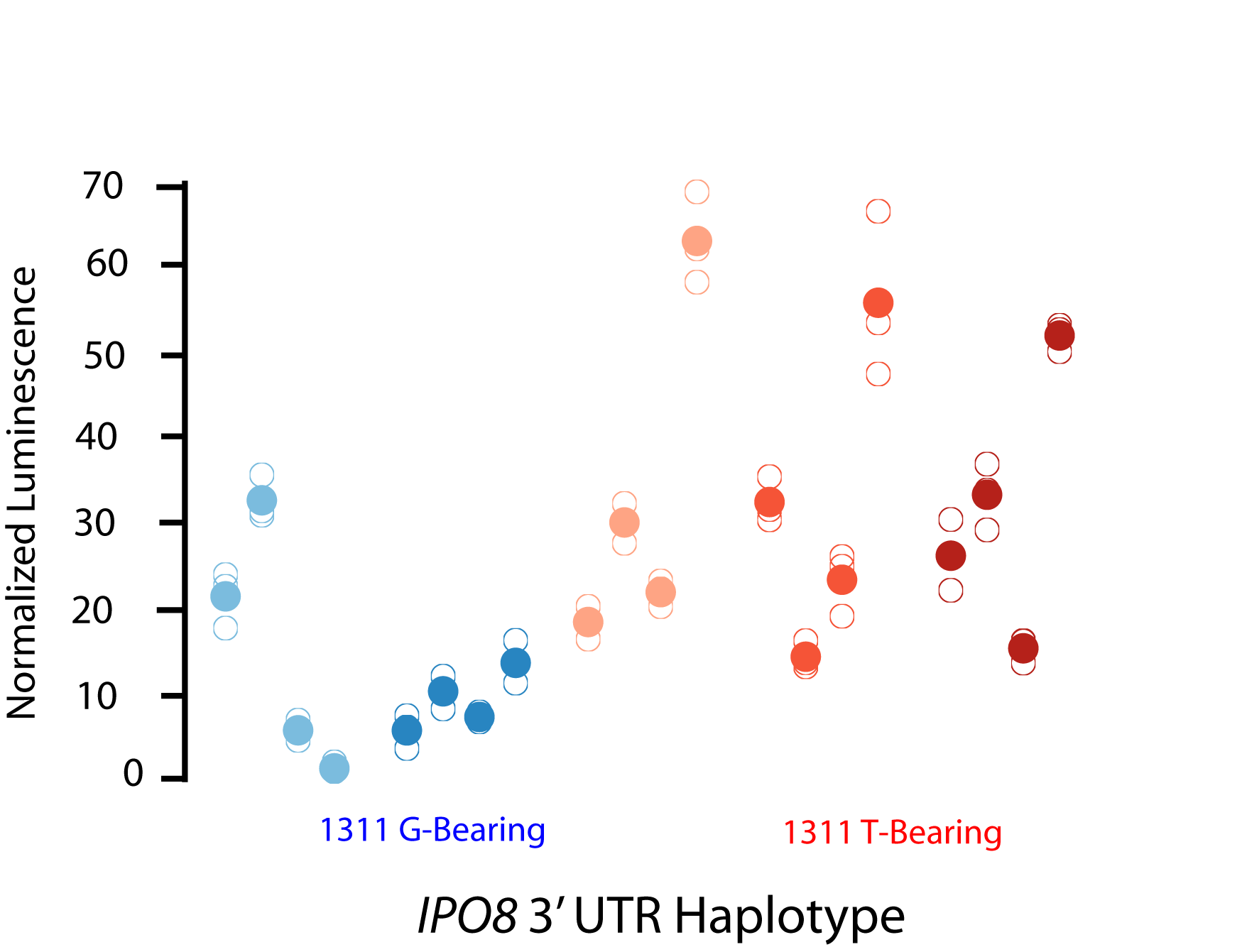

Supplement: Figure S12 — Replication of IPO8 3′ UTR expression effect. Reporter construct clones from each 3′ UTR haplotype were prepared and transfected independently of the data presented in Figure 5. Data depicted as in Figure 5, bottom panel. (TIF) [file pgen.1002078.s012.tif]
